# Peculiarities of Bacterial Cellulose

**DOI:** 10.3390/polym18111271

**Published:** 2026-05-22

**Authors:** Jiří Militký, Mohanapriya Venkataraman, Şebnem Sözcü

**Affiliations:** Department of Material Engineering, Faculty of Textile Engineering, Technical University of Liberec, Studentska 2, 46117 Liberec, Czech Republic; jiri.militky@tul.cz (J.M.); sebnem.sozcu@tul.cz (Ş.S.)

**Keywords:** bacterial cellulose, plant cellulose, waste, no-waste, drying methods

## Abstract

Cellulose is the most abundant renewable biopolymer, with bacterial cellulose (BC) emerging as a high-purity, sustainable alternative to plant-derived cellulose. While sharing the same chemical formula, BC possesses unique morphological characteristics, including a 3D nanofibrillar network, high crystallinity (>95%), and superior water-holding capacity (>60%), and is free of lignin and hemicellulose impurities. This review systematically explains the production, morphology, and properties of microbial cellulose produced by strains such as *Komagataeibacter*. We examine the influence of substrate composition, environmental growth conditions, and post-treatment protocols on the macro- and nanoscopic properties of the final pellicle. Furthermore, we discuss the high-performance applications of BC in medicine and health promotion, focusing on its efficacy as a wound dressing, artificial skin, and drug-delivery vehicle. Finally, current challenges in large-scale production and future strategies for tailoring BC properties are addressed.

## 1. Introduction

Our society is currently focused on developing multifunctional advanced materials from sustainable resources, such as cellulose. However, the traditional production of highly purified cellulose from plant sources requires removing side components (mainly lignin, hemicellulose, and pectin), which is energy-intensive and generates significant chemical waste that requires costly treatment. Consequently, achieving high-purity cellulose through environmentally benign routes remains a major research focus [1].

This review examines the production, properties, applications, and functionalization of bacterial cellulose (BC), a biofilm-forming material synthesized by selected bacteria. BC may serve as an alternative to conventional plant-derived cellulose while also providing enhanced characteristics that support its use in medicine, biomedicine, and health-related applications. BC is a natural, high-purity, sustainable biomaterial synthesized by anabolic reactions in some microorganisms, in the form of a pure cellulosic nanofibrillar network, used frequently for high-value medical and healthcare applications. BC is characterized by 3D ultrafine nanofibrous segments (20–100 nm in diameter), high crystallinity (84–89%), high porosity, excellent permeability, high water content, hydrophilicity, large specific surface area, low density, high purity, biocompatibility, and biodegradability [2]. BC can be prepared as a compact layer, a gel, a porous structure, or a fine dispersion [1].

Despite these advantages, many conventional BC manufacturing and processing methods rely on synthetic additives, expensive carbon sources, or chemical alterations, raising questions about sustainability, cost, and environmental impact. Consequently, research efforts have increasingly focused on developing greener and more economical BC fabrication strategies that preserve performance while reducing ecological burden [3,4]. Despite extensive research on BC, serious gaps remain in understanding and control structure–property relationships due to both bio-based and processing variability. Even under controlled cultivation (e.g., temperature, humidity, cell density, and metabolic efficiency), minor fluctuations of microbial activity can modify fibrous segments formation and final BC network architecture. These factors limit direct comparison of different studies, particularly when different growing conditions, bacterial strains, and processing routes are employed. Furthermore, most published studies focus on basic or limited drying methods. Systematic investigations linking drying strategies and pre-freezing techniques remain scarce. In addition, regenerating BC as an additive-free processing route is still insufficiently explored.

Consequently, the resulting material properties may also exhibit slight variability despite identical processing parameters [5]. By systematically examining how processing routes influence the resulting porous structure while accounting for these inherent biological variations, a scalable, industrially relevant pathway aligned with green chemistry and circular economy principles can be developed, contributing to the broader goal of sustainable material development while preserving environmental integrity [6].

The characterization techniques demonstrate that processing conditions strongly influence BC performance, particularly for potential biomedical, insulation, and filtration applications [4].

The main objective of this review is to analyze the key factors that influence BC morphology and properties, including raw materials, preparation methods, and post-processing steps, particularly water-removal procedures such as drying. The main areas of BC application and functionalization are presented.

## 2. Cellulose

**Figure 1:**
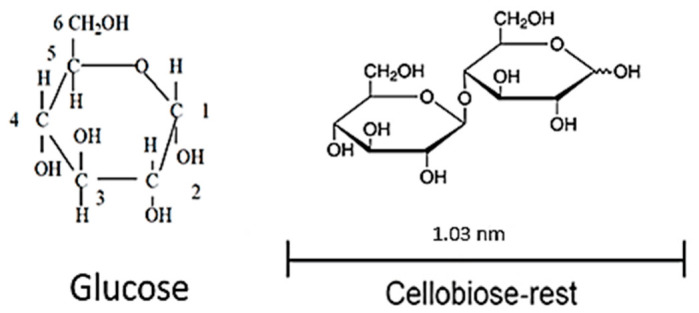
Structure of cellulosic rings (1.03 nm is size of the cellobiose-rest).

Cellulose is the most abundant renewable biopolymer on Earth. Chemically, it is a polyalcohol with one primary and two secondary –OH groups (see Figure 1).

In the pyranose ring, secondary hydroxyl groups are present at C2 and C3, whereas the hydroxyl group at C6 is primary [7]. The reactivity of these groups is in the order C6 >> C2 > C3. Among these groups, the hydroxyl group at C2 is the most accessible. The polymer backbone contains ether bonds, also referred to as glycosidic linkages, represented by –C–O–C–. The −OH groups are sites for hydrogen bonding and for some chemical reactions, such as esterification or oxidation. Oxidation at the hydroxy groups of cellulose can proceed in the C6 position via an aldehyde group to a carboxyl group and in the C2/C3 position to keto groups or (in the case of bond scission between C2 and C3) to the corresponding dialdehyde, oxidizable to the diacid, or reducible to the dialcohol [7]. Carboxyl groups are already intermediate-strong acid groups. They are dissociated roughly 1000 times faster than –OH groups. The result is an increase in cellulose’s negative charge.

Cellulose chains are connected by various systems of hydrogen bonds, which are responsible for the limited solubility in most solvents, the swelling in water, the reactivity of the hydroxyl groups, and morphological features (crystallinity) [8]. Cellulose also contains hydrophobic regions (around the C atoms) that partially influence overall solubility. Intermolecular hydrogen bonds are responsible for the strong interaction between cellulose chains. These bonds are produced between adjacent cellulose macromolecules located along the (002) plane in the crystal lattice of cellulose I (native cellulose), mainly between the oxygen atom in C3 and the −OH at C6 [7]. Cellulose has traditionally been obtained from plants, but it can also be produced through bacterial fermentation as an extracellular material generated by microorganisms. Although both materials are composed of linear β-(1,4)-linked D-glucopyranose units, their structure and physical behavior differ markedly because they arise from different biological systems [3,9,10,11]. In plant cells, cellulose is usually arranged hierarchically from clusters of chains, through micro and macro fibrils to fibrillar bundles, usually separated by a middle lamella. Microfibrils are the basic units of cellulose cell wall architecture. The thickness of bacterial cellulose microfibrils is around 4–7 nm, and plant cellulose microfibrils are thicker. For cotton linters, it is 7–9 nm; for ramie, 10–15 nm; and for dissolving pulp, 10–30 nm [7].

Many plant materials have a fibrous structure in which the length is roughly three orders of magnitude greater than the thickness. Similarly, fibrous materials are contained in woody plants. Their principal component is cellulose (α-cellulose), accompanied by other constituents such as hemicelluloses, lignin, and pectin. These structures can be considered composites of cellulose fibrils held together primarily by an amorphous matrix. The amorphous matrix phase in a cell wall is very complex and consists of hemicellulose, lignin, and, in some cases, pectin [12].

Hemicelluloses are characterized by low molecular chains composed of hexoses, pentoses, and parts of uronic acids. Single chains also contain D-xylose portions. Branched portions consist of both a D-xylose component as well as components of glucuronic acid and the corresponding methyl ester. They exhibit considerable chain branching with pendant side groups, giving rise to their noncrystalline nature. The degree of polymerization (DP) of hemicellulose is very low (50–200). This is 10–100 times lower than native cellulose [12].

Pectin is characterized by a high content of glucuronic acid and the corresponding methyl ester, and partially also the acetyl ester. Component D-galacturonic acid is combined with D-galactose and L-arabinose. They give plants flexibility. Pectin can be readily removed in alkalis, and it is sensitive to microbial attack and to enzymes (pectinases).

**Figure 2:**
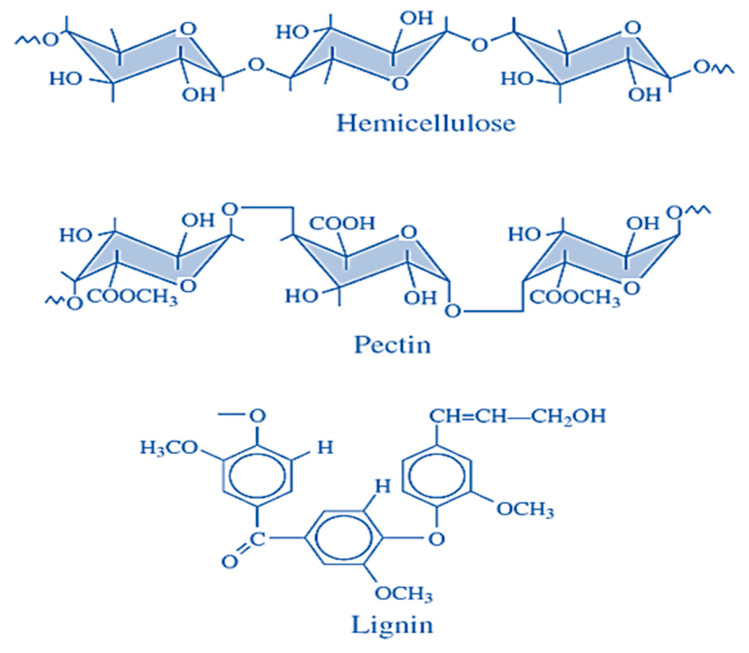
Schematic formulas of the side components of cellulose in plants.

Lignin is a complex thermoplastic hydrocarbon-based 3D copolymer with both aliphatic and aromatic constituents. Hydroxyl, methoxy, and carbonyl groups have been identified. Lignin is fully insoluble in most solvents and cannot be broken down to monomeric units. Lignin is totally amorphous and hydrophobic in nature [12]. The schematic structure of these constituents is shown in Figure 2.

The electrostatic interaction between cellulose and hemicellulose ranges from 38 to 57 mJ m^−2^. These forces between cellulose and lignin are much higher, i.e., 58 mJ m^−2^. The stronger lignin adhesion to cellulose is a result of the higher van der Waals energies [12]. Typically, in plants, the ultimate fibers are parts of bigger fibrous bundles as elements of so-called technical fibers. Individual technical fibers are composed of elementary fibers, usually glued together with pectin or lignin. Separation of cellulose from plants requires intensive chemical processing to remove impurities like pectin, xylan, lignin, and hemicellulose.

**Figure 3:**
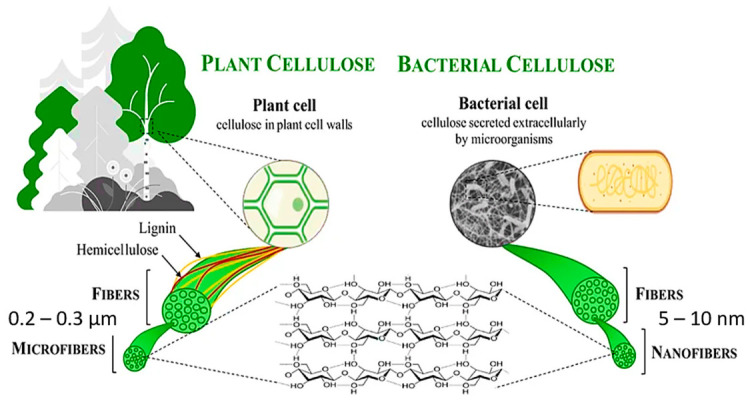
Comparison of the origin and structure of plant and bacterial cellulose [13].

The synthesis of bacterial cellulose, on the other hand, results in a pure cellulosic nanofibrillar network [13]. Bacterial cellulose (BC), a source of pure cellulose, is frequently used for high-value applications [14]. The main differences between plant cellulose and bacterial cellulose (see Figure 3 and Table 1) have been extensively discussed in the literature (e.g., [3,4,15,16,17]).

While plant cellulose is produced at a massive scale (1.5 × 10^12^ tons annually), bacterial cellulose production remains costly at an industrial scale. Current research focuses on optimizing fermentation media and identifying high-yield strains, such as *Komagataeibacter xylinus*, to make the process commercially competitive [4,11,18,19].

## 3. Bacterial Cellulose

BC is formed as a biofilm from substances secreted by specific bacteria during fermentation. The Fourier transform infrared (FTIR) spectrum of bacterial cellulose measured under ambient temperature shows distinct absorption bands that align with the chemical structure of cellulose and affirm the integrity of the cellulose backbone following ambient drying [20]. An extensive absorption band in the range of 3200–3500 cm^−1^ is ascribed to O–H stretching vibrations. This prominent signal indicates the significant hydrogen bonding inside the cellulose matrix, together with contributions from residual bound water retained in the material post-drying. Robust hydrogen bonding is characteristic of cellulose materials and signifies the close packing of fibrils resulting from capillary-induced densification during ambient drying. The absorption band at 2900 cm^−1^ is caused by the stretching vibrations of the C–H bonds in the aliphatic –CH and –CH_2_ groups that make up the cellulose molecule structure. The presence of this band shows that the polysaccharide backbone is still intact.

### 3.1. Bacterial Cellulose Peculiarities

**Figure 4:**
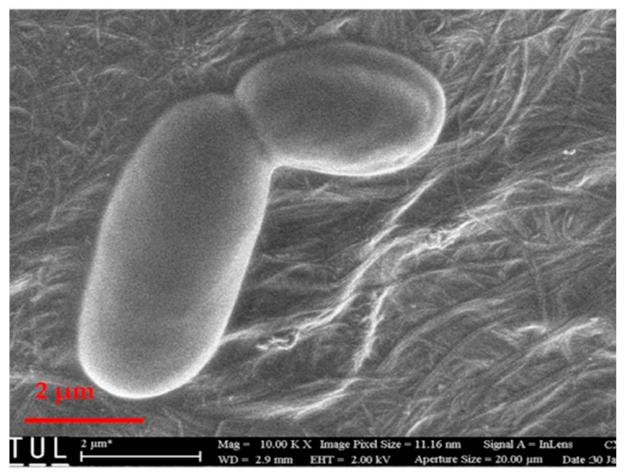
SEM image of bacteria on BC biofilm [22].

BC is a high-purity, sustainable biomaterial synthesized via anabolic reactions by certain microorganisms [15]. These microorganisms include certain algae and some Gram-positive and Gram-negative bacteria [21]. The genera *Komagataeibacter* and *Novacetimonas* produce large amounts of BC and are tolerant of low pH. Generally, BC forms a biofilm around bacteria, protecting them from water scarcity and damage from the environment (see Figure 4).

**Figure 5:**
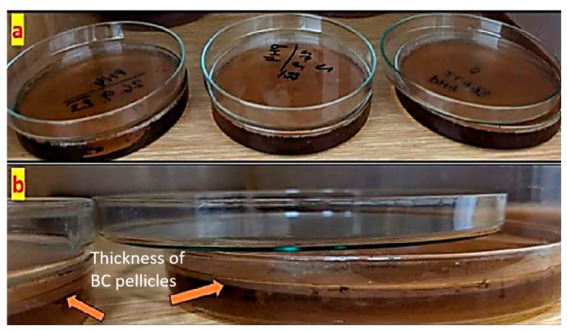
Synthesis of BC samples under static cultivation conditions. (**a**) Growth of BC pellicles in a Petri dish; (**b**) magnified view of BC pellicles with a thickness of approximately 2–3 mm [22].

Natural BC is synthesized from glucose through a sequence of enzyme-mediated reactions [15,23,24]. The microbial cell immediately secretes cellulose chains into the external environment, where they first self-assemble into fibrils and then into BC nanofibers. The presence of hydrogen bonds between the hydroxyl groups promotes the parallel stacking of cellulose molecules into crystalline nanofibers. These nanofibers, typically 25–100 nm in diameter and several micrometers long, assemble into a cellulose network that later develops into a dense, voluminous mat known as a pellicle [25]. BC-producing bacteria may be cultivated either in a static medium, which supports aerobic surface growth, or in an agitated medium, which generally produces irregular spherical pellets [26]; see Figure 5.

The distinctive features of BC include a three-dimensional nanofibrillar network, high crystallinity (84–89%), high porosity, excellent permeability, high water content, hydrophilicity, high specific surface area, low density, high purity, biocompatibility, and biodegradability [27]. These properties make BC a superior alternative for the medical, textile, and packaging industries [3,4,15,28,29,30].

BC usually contains only traces of microbial cells and their components, or the culture medium components. Purification is typically carried out using alkalis such as KOH or NaOH, organic acids such as acetic acid, or repeated washing in water. Bacteria synthesize cellulose by fermenting carbon and nitrogen sources. While traditional laboratory media use refined sugars (monosaccharides such as glucose and fructose, disaccharides like sucrose and maltose), researchers are increasingly utilizing industrial waste (waste beer yeast, whey from the dairy industry, and wheat thin stillage), agricultural waste (fruit juices and peels, coconut water, and molasses from the sugar industry), lignocellulosic resources (hydrolyzed wheat straw, rice bark, corn cobs, and coffee cherry husk) and nitrogen sources essential for bacterial growth (yeast extract, peptone, casein, and ammonium sulfate) [6,31,32,33]. A comparison of waste-derived and non-waste-derived BC is presented in Table 2, compiled from various articles [2,13,34,35,36,37,38,39,40,41,42].

Current research highlights that waste-derived BC can match the high purity (>99%), high water-holding capacity, and crystallinity of traditional BC while supporting a circular economy [6,41,43]. Key advancements include the use of agro-industrial waste (fruit peels, molasses, olive oil wastewater) and industrial by-products (crude glycerol) as cheap carbon and nitrogen sources [33,44,45].

### 3.2. Bacterial Cellulose Production from Non-Waste Sources

BC production from non-waste sources typically uses specialized laboratories or industrial-grade culture media. Unlike waste-based production, which uses agricultural or food scraps, non-waste production utilizes refined sugars and nutrients to ensure high purity and consistent structural properties [22,46,47,48,49,50]. BC yields typically range from 1.13 g/L to 2.0 g/L under standard conditions. Its advantages include consistent quality, a predictable fiber diameter of about 38 nm, and controllable crystallinity. The main limitation is the high cost of commercial substrates. The most effective bacteria for synthesizing BC are acetic acid bacteria [4,51], such as *Komagataeibacter xylinus* (formerly *Acetobacter xylinum*). The most common non-waste medium is the Hestrin and Schramm (HS) medium [52,53,54,55].

The method of bacteria growth (static or agitation) determines the physical form of the resulting BC [9,42,56,57]. To increase BC production and develop high-value applications, strategies that focus on optimizing fermentation parameters, utilizing low-cost substrates, and employing genetic engineering to improve strain efficiency are proposed [57,58,59,60].

### 3.3. Bacterial Cellulose Production from Waste

Using waste materials for BC production can substantially lower costs, since BC produced with synthetic media is often far more expensive than plant cellulose [6,32,47,61,62].

Various industrial and agricultural residues are effectively repurposed as low-cost substrates for BC production [9,33,55,63,64,65]. Recent research shows that using waste streams such as molasses, cheese whey, fruit peels, and crude glycerol effectively replaces conventional media, yielding significant BC amounts while promoting a circular bioeconomy [40,43,49,66,67]. BC obtained from waste sources, including agro-industrial residues, food waste, and industrial by-products, generally shows structural and physicochemical properties that are comparable to, and in some cases better than, those of BC from non-waste sources [2,6,13,34,35,36,37,38,39,40,41,42]. Waste-based bacterial BC requires more processing than non-waste bacterial cellulose to prepare the feedstock. However, it is important to note that the downstream processing (purification) of the final cellulose is similar for both, and the overall industrial “processing” of waste is often considered part of a sustainable, cost-effective, and environmentally friendly cycle [68,69].

Drying BC from both non-waste and agricultural/industrial waste media focuses on optimizing specific structural, mechanical, and functional properties while improving energy efficiency. Because native BC is over 95% water, the drying process is critical, as it directly determines whether the final product is a dense film or a porous aerogel [5,70,71]. Current research highlights that the drying method is more influential on final material properties than the feedstock origin (waste vs. non-waste) [48,72]. Non-waste BC is typically freeze-dried, especially for high-end biomedical applications. Waste-derived BC can be oven-dried or freeze-dried after proper purification.

The drying parameters, therefore, play an important role in determining how the initial nanofibrillar network evolves during processing, ultimately controlling pore structure and density. High-pressure homogenization prior to freeze-drying has been used to increase the crystallinity of waste-derived BC compared to standard oven drying. The morphological changes in BC associated with different drying methods are summarized in Table 3. A comprehensive comparison of basic drying methods was published [1] with these main findings:

- **Room-temperature drying**: Capillary forces caused marked structural densification. SEM analysis showed fibril aggregation and pore reduction, resulting in relatively high density (~0.63 g/cm^3^) and moderate porosity (~59.4%). Despite structural collapse, cellulose chemistry remained unchanged, as confirmed by EDX (C and O dominance) and FTIR, which preserved cellulose I [7].
- **Supercritical CO_2_ (ScCO_2_) drying**: Minimization of capillary stresses preserved the nanofibrillar network. SEM revealed a highly porous isotropic structure (~46–56 nm fibers), while BET analysis showed the highest surface accessibility (~123–124 m^2^/g, pore volume ~0.35–0.36 cm^3^/g). The BC exhibited ultralow density (~0.01 g/cm^3^) and >99% porosity, though thermal conductivity (~0.040–0.042 W·m^−1^·K^−1^) remained slightly higher than the freeze-dried samples.
- **Freeze-drying**: Pore architecture was governed by ice-templating during pre-freezing. Freezing at −18 °C produced heterogeneous pores, whereas liquid nitrogen freezing produced more homogeneous, interconnected networks. In situ freezing in a lyophilizer enabled additive-free porous BC cryogels, although environmental conditions during drying influenced structural reproducibility.

Among all these routes, the cryogels exhibited the best thermal insulation performance, reaching the lowest thermal conductivity of 0.030 W·m^−1^·K^−1^. In comparison, ScCO_2_-dried aerogels and regenerated BC samples showed higher conductivities (~0.040–0.042 W·m^−1^·K^−1^). This increase in regenerated BC is attributed to fibrillar reorganization and partial densification during mechanical disintegration and subsequent drying [20].

### 3.4. Properties of Bacterial Cellulose

**Figure 6:**
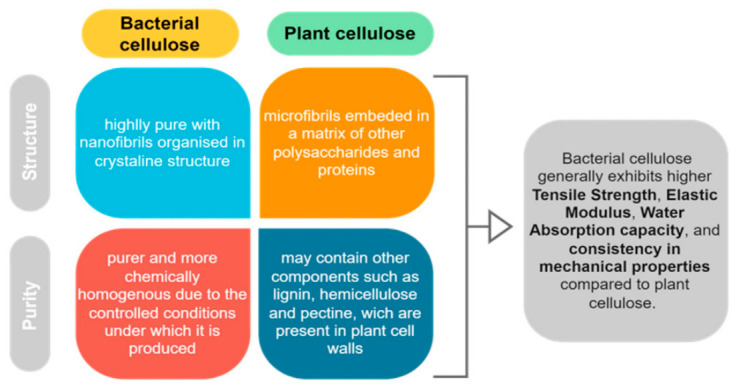
Comparison of plant-based and bacterial cellulose [74].

Recent studies indicate that the physical properties of BC depend on the nutrient source used during production [40,73,74,75,76]. BC from non-waste media is typically “ultrapure,” whereas waste-derived BC may require more rigorous post-production cleaning to remove residual pigments or proteins from the complex waste substrate. Comparison of plant-based and bacterial cellulose is shown in Figure 6.

Crystallinity of BC generally ranges from 74% to 96% for both sources. Some waste substrates (like fruit juices) can slightly lower crystallinity compared to pure glucose media due to the presence of non-sugar organic compounds. Waste-derived BC has tensile strength between 72 and 140 MPa, while non-waste BC often reaches 200–400 MPa. The initial modulus of waste-derived BC typically falls within the range of 0.97 to 1.64 GPa, compared to up to 15–18 GPa for non-waste BC.

Waste-derived BC shows excellent water holding capacity, often between 102 and 138 g water/g dry BC, which is comparable to or occasionally higher than non-waste BC due to higher porosity in certain waste-based fibril networks [32,40,75,76,77,78,79]. Polymerization degree of BC from non-waste media typically ranges from 2000 to 6000, while waste-derived BC can vary more significantly depending on the specific industrial byproduct used [15,40,80].

### 3.5. Advantages and Limitations of BC

BC is a versatile, non-toxic, and biocompatible material suitable for wound healing, tissue engineering, and drug delivery because it combines high water retention (>90%) with exceptional purity. Key advantages include moldability, non-adherence to wounds, and structural similarity to collagen. Limitations include high production costs, low biodegradability in the body, and a lack of innate antimicrobial properties [81,82,83,84,85]. Enhancing BC functionality can be achieved in different ways. Drying with supercritical CO_2_ can be used to adjust the crystallinity and mechanical behavior of BC. By using templating techniques (introducing porogens as paraffin spheres), the BC pores are in the 300–500 µm range, compared with standard BC pores in the 0.02–10 µm range. Templating then improves cellular infiltration.

Generally, drying conditions critically govern open-pore preservation and nanofibril aggregation, strongly affecting mesoporosity and thermal transport.

Combining BC with natural or synthetic polymers (e.g., chitosan, gelatin, silk-sericin, and alginate) overcomes its low perpendicular compression strength. TEMPO-mediated oxidation can make BC more biodegradable. In situ or ex situ incorporation of silver nanoparticles (AgNPs), ZnO, or antibiotics (e.g., cefoperazone) transforms BC into an antibacterial, infection-resistant dressing. A similar effect can be obtained by modifying the BC surface with quaternary ammonium compounds or other antimicrobial peptides [81,86,87,88]. Grafting bioactive molecules, such as hydroxyapatite, onto the BC scaffold enhances osteoconductivity for bone tissue engineering. Using gelatin as a surface modifier mimics the properties of natural collagen, thereby improving cell adhesion and proliferation [81,85,89].

### 3.6. Applications and Modifications of BC

**Figure 7:**
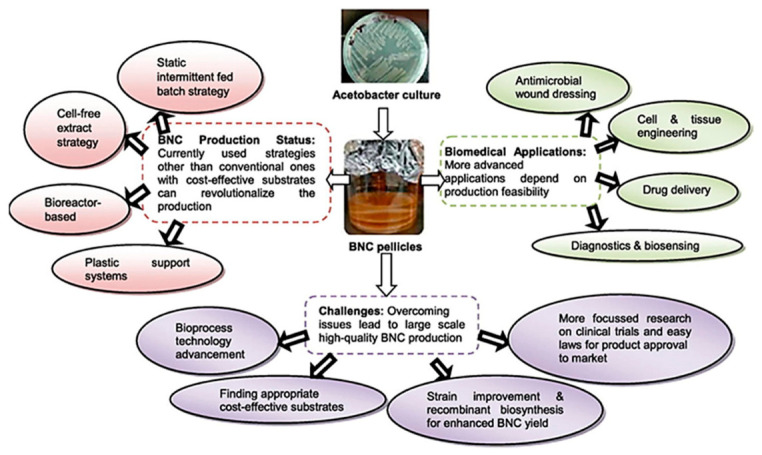
Recent applications of BC membranes in different fields [50].

BC has become a highly developed material platform for medical and healthcare applications, serving as a high-purity biomaterial for wound care, tissue engineering, and drug delivery [9,82,90]. Non-waste BC yields high-purity membranes, often used for high-end clinical applications such as corneal substitutes, vascular grafts, and neural implants. Waste-derived BC is increasingly prioritized for sustainable, large-scale production of wound dressings and healthcare packaging without sacrificing essential mechanical properties [83,84]. Some applications of BC membranes across different fields are shown in Figure 7.

Key natural additives include antimicrobial compounds (such as lemongrass oil and silver nanoparticles), proteins (such as collagen and gelatin), polysaccharides (such as alginate and chitosan), and plant extracts, which impart bioactive, antimicrobial, or regenerative properties [49,88,91]. BC membranes can function as a “second skin” for severe burns and chronic ulcers by preserving a moist environment and encouraging cell migration. The three-dimensional nanofibrillar architecture of BC resembles the human extracellular matrix, supporting the growth of bone, cartilage, and dental implants. BC serves as an efficient carrier for topical and oral medications [92].

**Figure 8:**
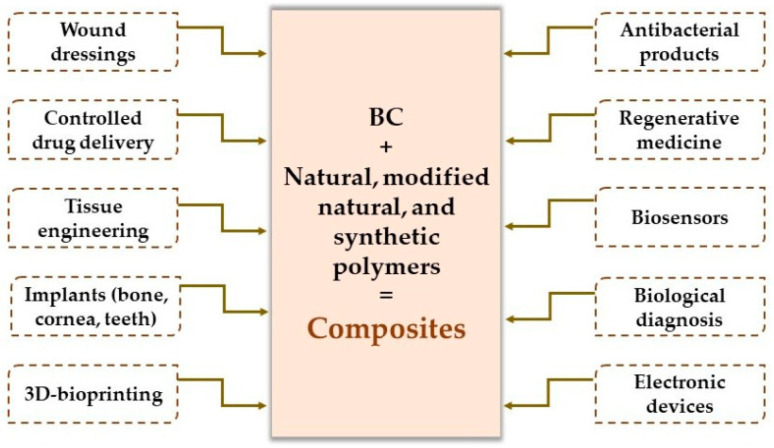
The major applications of bacterial cellulose-based composites [31].

Oxidized BC scaffolds impregnated with chitosan and collagen peptides for potential implant tissue engineering are proposed [93]. Recent studies highlight its use for delivering anticancer therapeutics with reduced systemic toxicity and antibacterial nanoparticles (e.g., silver) to combat resistant infections. Emerging state-of-the-art applications of BC include artificial blood vessels, urethral and nerve grafts, and retina implants, due to its low tissue adhesion and high mechanical strength [18,31,50,82,88,94,95,96]. It is increasingly integrated into bio-inks for 3D printing complex, patient-specific anatomical structures. BC is also used for emerging non-medical applications. To overcome a lack of inherent antimicrobial activity, BC is now frequently combined with nano polymers, plant extracts, or bioactive glass. BC-based hydrogels and biofilms are being explored for use in wearable biomedical devices and diagnostic monitoring systems [18,31,88,96]. The major applications of bacterial cellulose-based composites are illustrated in Figure 8.

## 4. Conclusions

Cellulose is the most important biopolymer due to its abundance, renewability, mechanical robustness, biocompatibility, hydrophilicity, and thermal stability, and is widely used for various purposes, such as textile materials, packaging materials, composite reinforcements, and geocomposites. Recently, cellulose has been adapted for advanced material applications. Research has expanded its use into pharmaceutical formulations, membrane and filtration systems, drug delivery platforms, and as a stabilizer or emulsifier. For these applications, plant-derived cellulose may be replaced by bacterial cellulose, which can also be produced from a range of waste streams. Even when cultivation parameters differ only slightly, variations in microbial activity and local growth conditions can affect fibril formation and the development of the initial nanofibrillar network. The biological changes resulted in slight structural differences in the generated membrane-like particles, thereby influencing the material’s response to the drying procedures. As a result, small differences may still appear in the final microstructure and measured material properties, even when identical drying and characterization procedures are used. Therefore, when selecting applications for BC-based materials, both biological variability and processing-related effects should be considered carefully because they may influence structural and functional performance.

Various functionalization approaches have been developed to introduce reactive groups, alter surface energy, and improve compatibility with polymer matrices. Among the most widely studied modifications are acetylation, oxidation, plasma-assisted functionalization, and catalytic surface reactions [23].

## Figures and Tables

**Table 1:** Key Comparison: Bacterial vs. Plant Cellulose [3,4,15,16,17].

Feature	Bacterial Cellulose (BC)	Plant Cellulose (PC)
Purity	Extremely high (no lignin/hemicellulose)	Low (requires chemical removal)
Structure	3D reticulated nanofiber network	Microfibrillar bundles
Strength	High tensile strength and crystallinity	Variable; generally lower crystallinity
Hydration	High water retention (up to 100× weight)	Moderate to low

**Table 2:** Comparative analysis of waste and non-waste-derived BC.

Feature	Waste-Derived BC (e.g., Fruit Peels, Molasses)	Non-Waste BC (e.g., Glucose, HS Media)
Cost	Very low; utilization of wastes	Higher commercial sugars/nutrients are more expensive.
Sustainability	High; supports waste treatment and reduces pollution	Lower, dependent on the agricultural products.
Crystallinity	Often higher (77.39% for vegetable waste)	Relatively high (66.88%)
Consistency	Dependent on waste composition and pretreatment, as hydrolysis	Standard nutrient concentrations
Yield Potential	Often 4–6 times higher than HS medium Range 13–30 g/L (based on substrate)	Stable but lower than optimized waste-derived (1.1–2.0 g/L)
Pre-treatment	Commonly by hydrolysis or sterilization	Not necessary; ready for fermentation

**Table 3:** Comparison of drying methods.

Method	Product State	Structural Effect	Best Application
ScCO_2_ Drying	Aerogel	Preserves nanopores	Tissue engineering, super-insulation
Freeze-drying	Cryogel	High porosity, light	Biomedical, drug delivery
Heat-press	Dense Film	Dense, flat, high crystallinity	Textiles, electronics, packaging
Microwave	Dry Sheet	Smooth, Energy Efficient	Rapid production, bio-packaging
Oven Drying	Dense Film	Shrinkage, high density	Basic material production

## Data Availability

All data relevant to this study are included in the manuscript.
